# Cardiovascular Disease Risk Factors in the Native American Population

**DOI:** 10.3390/jcdd12010027

**Published:** 2025-01-16

**Authors:** Krista Goerger, Karla Abbott, Mark K. Larson, Michael Holinstat

**Affiliations:** 1 Department of Pharmacology, University of Michigan, Ann Arbor, MI 48109, USA; kgoerger@umich.edu; 2 Nursing Department, Augustana University, Sioux Falls, SD 57197, USA; karla.abbott@augie.edu; 3 Biology Department, Augustana University, Sioux Falls, SD 57197, USA; mark.larson@augie.edu

**Keywords:** cardiovascular disease, Native American, American Indian, under-represented, chronic stress

## Abstract

Native Americans are disproportionately affected by cardiovascular disease in comparison with other racial and ethnic groups in the United States. Previous research has analyzed risk factors, quantified prevalence rates, and examined outcomes of cardiovascular disease in Native Americans, yet few studies have considered the role of societal and psychological factors on the increased burden of cardiovascular disease in Native Americans. Modifiable risk factors for cardiovascular disease, including poor nutrition, reduced physical activity, obesity, and increased substance use, are exacerbated in Native American communities due to cultural and historical factors. Further, Native Americans have endured historical trauma and continue to experience additional financial and healthcare stressors, resulting in increased levels of chronic stress. Chronic activation of stress responses through the hypothalamic–pituitary–adrenal and autonomic nervous system increases inflammation and cardiovascular dysfunction resulting in an increased risk for cardiovascular disease. Therefore, it is critical to examine the connection between these stressors and the cardiovascular health disparities in Native American communities to create effective strategies to improve health outcomes.

## 1. Introduction

Cardiovascular disease (CVD) used to be rare in the Native American population and it was thought Native Americans had inherent protection from CVD [1,2]. However, studies in recent decades have shown CVD rates continue to climb [3], and it is well-established that CVD is now the leading cause of death in Native American populations [4]. While CVD poses a significant health risk for the entire U.S. population, it is important to acknowledge the increased burden the Native American population faces. Native American and Alaskan Native populations are disproportionately affected by coronary heart disease (CHD) with a prevalence rate of 12%, which is two-fold higher compared to the U.S. population [2,5,6]. In 2018, Native Americans were 10% more likely to have a stroke compared to non-Hispanic white adults [7]. In addition, overall cardiovascular events among Native American populations are 20% more fatal [8]. There has been significant research conducted to assess the prevalence of CVD among Native Americans, but few studies have considered the role cultural and psychological factors such as Westernization, long-term neglect, and mistreatment play in the increased burden of CVD. Further, it is difficult to fully understand the various CVD risk factors present in Native American populations due to the variation between communities and tribes. There are over 500 federally recognized American Indian and Alaska Native tribes with many different traditions, beliefs, and histories [9]. While these differences should not go unnoticed and more research is needed to thoroughly consider the relationship between the culture and health within each of the communities, for the purpose of the review there will be some cultural generalizations made due to the lack of scientific research available.

## 2. Cultural Influence on Classic Cardiovascular Risk Factors

The well-established, classical modifiable cardiovascular risk factors include, but are not limited to, eating non-nutritious food, low levels of physical activity, smoking, and drinking [10]. While these risk factors transcend all people irrespective of age, gender, or geographic origin, some of these modifiable factors are more prominent in Native American communities due to cultural and historical factors. Pinpointing how these factors are integrated into Native American communities is key to ameliorating their effects and can inform the development and improvement of culturally relevant programs to encourage healthier habits and reduce the risk for CVD.

### 2.1. Nutrition

Prior to Western European expansion into Native American land and assimilation into the Western European diet, Native Americans acquired their dietary needs through hunting wild game, fishing, and harvesting native plants such as corn, beans, squash, seeds, nuts, and fruits [11]. When Native Americans were relocated from their native lands to reservations, they were forced to change their diet abruptly. The traditional food they had grown accustomed to was no longer available, resulting in an increased reliance on the U.S. government for rations. In 1871, these rations typically consisted of fresh beef, bacon, flour, corn meal, coffee, salt, sugar, and rice, which discouraged the consumption of nutritionally diverse foods and promoted the consumption of high-caloric foods with few nutritional benefits [12]. Today, many Native Americans continue to depend on the dietary food sources provided by government Commodity Supplemental Food Programs [13]. Like rations, commodity food often consists of calorie-dense, processed foods such as sugar, enriched flour, vegetable shortening, canned meat, cheese, peanut butter, sugared juices, powdered milk, and corn syrup [13].

Another factor contributing to the highly processed diet is limited access to nutritious foods, as there are fewer sources of healthy food per square mile on tribal land compared to non-tribal land [14]. When interviewed, many Native Americans expressed that the expense of nutritious foods and the transportation required to access nutritious foods were significant barriers to eating healthy [14]. The overall shift in nutrient intake is evident within the Pima Indian community residing in southern Arizona. It is estimated the traditional Pima Indian diet from over 130 years ago consisted of ~70–80% carbohydrates, ~8–12% fat, and ~12–18% protein [15], while a 1996 survey indicated their diet consisted of ~48% carbohydrates, ~34–36% fat, and ~15% protein [16]. The Strong Heart Study (Phase II) conducted with participants from 13 tribes supports these findings, as their results showed similar estimates of fat intake at around ~35% [17].

Processed foods often contain high amounts of sodium, and a 2005 report demonstrated Native American participants’ intake elevated sodium levels compared to national recommendations [17]. Sodium can contribute to CVD risk by increasing blood pressure or hypertension. However, a 2023 report stated Native American adults were 10% less likely to have high blood pressure [18], suggesting sodium intake and hypertension do not contribute to the increased burden of CVD in Native American populations.

Diets of highly processed food also contain high levels of saturated fat [19,20], which is linked to an increased risk of CVD [21,22]. This is due to the development of dyslipidemia, or an unfavorable lipid profile containing high levels of low-density lipoprotein cholesterol (LDL-C) and triglycerides (TG), and lower levels of beneficial high-density lipoprotein cholesterol (HDL-C) [23,24]. Unfavorable lipid profiles can result in the buildup of plaque in the major arteries, a CVD also known as atherosclerosis [25]. In 2005, Native American participants demonstrated elevated intake of cholesterol and energy from total fat compared to national recommendations [17]. Further, a recent update from the Strong Heart Family Study found more than 70% of Native American young adults and 50% of Native American teens have dyslipidemia [26]. However, other studies have found cholesterol levels are not elevated in Native American adults compared to non-Hispanic white adults or the U.S. average [18,27]. Therefore, future studies need to continue to determine the prevalence of dyslipidemia in the Native American population and the link between consumption of highly processed foods.

Consumption of processed foods also results in the inadequate consumption of fruits and vegetables, resulting in a deficiency in essential vitamins including vitamin A, vitamin C, and folate relative to national recommendations [17]. A more recent survey further supports these original findings, in which 56% and 75% of Native American adults reported low fruit and vegetable intake, respectively [28]. Studies have shown that a decreased intake of fruits and vegetables increases the risk of CVD [29,30], and while the exact mechanisms of the ability of fruits and vegetables to reduce CVD risk is unknown, it is believed to be due to their antioxidant and anti-inflammatory properties resulting in reduced blood pressure [31,32].

The transition from a traditional diet to processed foods is strongly associated with poor cardiovascular health and may play a significant role in the increased risk of CVD among Native Americans. Although this dietary shift occurred several generations ago, limited access to nutritious foods continues to affect this population. However, diet alone does not fully account for the heightened CVD burden, as research indicates relatively minimal differences in nutritional intake between Native Americans and the general U.S. population. It is important to note that nutritional studies focusing on Native Americans have been conducted independently of those examining the broader U.S. population, complicating direct comparisons.

### 2.2. Physical Activity

Engaging in physical activity has long been observed to have beneficial effects on cardiovascular health [33], yet physical inactivity is a problem in all facets of the U.S. population, including Native American communities [34]. The results of several studies report only about ~20–30% of Native American adults and children engage in physical activity [35,36,37,38], and between ~30–50% of Native Americans are physically inactive [39,40]. There is a limited body of evidence comparing physical activity rates between Native American populations and the U.S. population, but one study claimed Native Americans are more likely to be physically inactive relative to other races [40]. Further, the National Health Interview Survey from 2018 reported that 18.7% of Native Americans met federal physical activity guidelines compared to 25.8% of non-Hispanic white adults [7]. Unfortunately, most of this evidence is based on physical activity data collected using self-reported subjective measures of physical activity, which often suffer from reporting bias [41].

The sedentary lifestyle of many Native Americans can in part be attributed to Western influence. In 2000, Pima Indians residing in Mexico were found to still live a traditional lifestyle with labor-intensive, non-mechanized farming and building, while U.S. Pima Indians in Arizona utilized mechanized farming methods and participated in more activities of lower intensity. U.S. Pimas showed significantly reduced levels of physical activity compared to age-matched Mexican Pimas [42]. Mexican Pimas are also lighter and have lower body mass indexes (BMIs) than U.S. Pimas, further supporting the importance of physical activity to protect against the development of CVD and obesity [43]. Other barriers, including inadequate exercise facilities, lack of access to transportation, unsafe walking and trail conditions, shortage of time outside work, and inclement weather, deter Native Americans from attaining normal levels of physical activity to maintain cardiovascular health [44,45]. Women are also more likely to be inactive compared to men [40], and according to a focus group interview this is likely due to insufficient support from the community to be physically active, a lack of support for household and childcare responsibilities, and difficulties balancing societal expectations with physical activity [45].

While more quantitative research is needed to fully understand the prevalence and factors contributing to physical inactivity across diverse Native American communities, existing evidence suggests that low rates of exercise, limited access to resources for physical activity, and societal pressures deprioritizing its importance likely play a significant role in the elevated rates of cardiovascular disease (CVD) within these populations. Recent studies have increasingly focused on interventional strategies, particularly targeting children and youth, to promote physical activity. However, these studies are often constrained by small sample sizes and reliance on self-reported data [46]. Interventions at the group, clinic, and individual home-based levels have demonstrated improvements in physical activity levels, but further research is necessary to broaden the application of culturally centered approaches, which may better address the unique needs of Native American communities [47].

### 2.3. Obesity

Obesity, defined as a BMI greater than 30, is a significant risk factor for CVD and is associated with several key contributors to CVD risk. These include an increased likelihood of hypertension, elevated levels of LDL-C and TG, and reduced levels of HDL-C [48,49,50]. Additionally, obesity is strongly linked to increased overall cardiovascular morbidity and mortality [48]. Consistent with high rates of CVD, obesity rates in Native American communities exceed all other racial and ethnic groups [50], and in 2018 the National Health Interview Survey found that 48.1% of Native Americans were obese compared to 33.9% of non-Hispanic whites [18]. Further, in communities surveyed in 2010, more than one-third of Native American adults were found to be obese, whereas nationally, one-fifth of U.S. adults were reported to be obese [51]. Additionally, Native American children are also at a high risk for developing childhood obesity, which is a major risk factor for adult obesity [52,53,54]. The prevalence rate of obesity in Native American preschoolers (31.2%) was found to be higher when compared to other racial/ethnic groups, including whites (15.9%) [52]. With obesity rates increasing at younger ages, obesity continues to be embedded into the population, exacerbating the burden of CVD.

Obesity is a multifaceted disease caused by biological, socioeconomic, and environmental factors resulting in energy intake exceeding energy expenditure. Two classic causes of obesity are poor nutrition and lack of physical activity. Therefore, the increased rate of obesity in Native Americans is anticipated, as studies have shown Native Americans consume more processed foods and engage in less physical activity than the general population.

### 2.4. Tobacco Use

Native Americans have a long history of ceremonial tobacco use. While the purpose of tobacco use varies widely across tribes, it was traditionally primarily for religious and therapeutic purposes, and today, it is still used for burial offerings or as a gift [55]. Although tobacco has cultural origins, its shift to recreational use has become a major source of addiction for Native Americans across the nation. The 2018 SAMHSA study reports Native Americans have higher rates of cigarette use in their lifetime compared to all other racial/ethnic groups [56]. Additionally, 43.3% of Native American adults used tobacco in the past month compared to 25.4% of whites [56]. Youth and adolescents also have increased rates of tobacco use compared to the overall U.S. population [57,58,59].

Cigarette smoking is a major cause of CVD and about one in three deaths from CVD in the U.S. are attributed to smoking [60,61]. The exact mechanisms linking cigarette smoking to related CVDs are poorly understood, but research has shown smoking cigarettes promotes mitochondrial oxidative stress, endothelial dysfunction, procoagulant states, and reduced HDL-C [62,63]. These alterations increase blood pressure, risk for thrombosis, and inflammation [64]. To combat the high rates of tobacco use in Native American communities and other minorities, several public health programs have been put in place [65]. Prevention and/or cessation interventions enlist elders, tribal leaders, parents, or school personnel to develop a curriculum to intervene with adolescents in a school-based setting [65]. In general, the prevention programs have reduced adolescent tobacco initiation rates but have not improved cessation rates [65,66]. Instead, it was found individuals who received a diagnosis of diabetes mellitus, hypertension, or renal insufficiency were more likely to quit tobacco use [67]. There is still a need to create more effective programs to reduce cigarette use in this high-risk population, and the programs must also address the rise of e-cigarette and smokeless tobacco products, which, despite being marketed as a safer alternative to cigarettes, are both linked to adverse health effects [68,69].

### 2.5. Alcohol Use

Prior to European colonization, Native Americans had minimal exposure to alcohol. However, with the widespread availability of alcohol introduced by European settlers through trade, recreational alcohol use increased rapidly among Native American tribes [70]. This historical shift has contributed to the enduring perception of high alcohol misuse within Native American communities. Yet, evidence suggests that Native Americans exhibit higher rates of alcohol abstinence compared to whites [71]. For instance, the 2018 SAMHSA study reported that lifetime alcohol use, as well as alcohol use in the past month or year, was lower among Native Americans than among whites across all age groups [56].

Despite lower overall alcohol consumption, lifetime rates of alcohol dependence in some tribal groups have been reported to range from 20% to 70%, far exceeding the epidemiological prevalence of DSM-IV alcohol dependence, which is 13% in the U.S. general population [72]. Additionally, episodic heavy drinking or binge drinking appears to be more prevalent among Native Americans [56,73]. One study found that 37% of individuals from the Cheyenne River Sioux Tribe in South Dakota had consumed more than five drinks on a single occasion at least once in the past month, compared to 29% of the general South Dakota population [73]. The high rates of binge drinking are concerning as binge drinking is correlated with increased risk for several CVDs, including thrombosis, hypertension, and sudden cardiac death [74].

One promising trend within Native American communities is an increase in the number of heavy-drinking men deciding to quit drinking without treatment programs. These men reported the main motivation for quitting was health concerns [75]. Additionally, there are higher rates of alcohol abstinence among Native American females [73]. These findings suggest that alcohol use within Native American populations may be on a gradual decline. Nevertheless, the persistence of alcohol abuse and binge drinking highlights the need for continued attention, as these behaviors likely contribute to the elevated prevalence of CVD observed in Native American communities.

## 3. Societal Stressors as Cardiovascular Risk Factors

Many classical cardiovascular risk factors can be eliminated with proper resources to support lifestyle modifications and access to health care. However, other risk factors constructed by society and the surrounding environment also play a large role in Native American cardiovascular disparities. Native Americans have endured many traumatic events since the colonization of the Americas and continue to experience discrimination at both individual and institutional levels [76]. Enduring mistreatment and oppression today, on top of years of generational trauma and stress, can have a large impact on overall health [77,78]. These stressors can result in physiological changes that have been shown to negatively impact cardiovascular health and increase the risk for CVD [79], demonstrating the importance of considering societal stressors when discussing the current state of Native American heart health.

### 3.1. Historical Trauma

Multi-generational physical, psychological, and cultural trauma experienced by the Native American communities have resulted in a longer-lasting composite stressor and is called “historical trauma” [9]. Historical trauma was defined by Maria Yellow Horse Brave Heart as “cumulative trauma over both the life span and across generations results from massive cataclysmic events” [9]. Throughout the history of interactions between Western Europe and the Native American nations, there has been a continual decline in the Native American population. Due to diseases carried by American settlers to North America and armed conflict, the Native American population declined rapidly from an estimated 12 million prior to European settlement to less than 300,000 in 1990 [80]. Discussions in Native American focus groups have shown many Native Americans across all ages often have thoughts about historical losses in their community, and these thoughts are accompanied by emotional distress [76].

The passage of the Civilization Fund Act in 1819 led to the creation of Indian boarding schools, forcing children to leave their homes to attend boarding schools [81]. The mission of these schools was to integrate the children into Western American culture, and they often discouraged children from speaking their native language, practicing their native religion, or engaging in any other native traditions [81]. In addition to the forced integration policies, children were often placed in physical and psychological danger, as evidenced by the numerous reports of sexual and physical abuse, malnutrition, child labor, neglect, and death [82,83]. One Lakota study found parents who attended boarding school as children felt like inadequate parents and were overwhelmed by their role. Subsequently, the children of adults who were raised in boarding schools more often reported abuse and neglect in their own childhoods and felt unprepared to raise their own children [84].

Based on these studies, trauma experienced from the rapid decline in population, assimilation policies, and boarding schools was likely passed down through multiple generations. One study has reported that early-life intrapersonal trauma and community family dysfunction were significantly associated with an increased risk of diabetes, a major risk factor for CVD [85]. Therefore, historical trauma is still reverberating in Native American communities today and affecting overall health, including the risk for CVD.

### 3.2. Socioeconomic Status and Financial Strain

According to a 2022 poll from NPR and Harvard, Native Americans experience more financial strain compared to any other racial or ethnic group in the U.S. [86]. Nearly 40% of Native Americans struggle to afford food compared to 21% of white adults, and a majority (58%) of Native Americans do not have enough emergency savings to cover one month of expenses. In 2010, 23% of Native American families reported an income level below poverty as defined by the U.S. government, compared to 16% of the general U.S. population [87]. The increased poverty rates of Native Americans can be largely attributed to a lack of jobs and low wages [88], as the unemployment rate of American Indians in 2022 averaged 6.2%, more than any other racial group [89].

Both increased financial strain and low socioeconomic status have been shown to increase the risk for CVD [90,91]. This is largely because people with low socioeconomic status and increased financial strain are less likely to eat nutritious food [92,93] and are less likely to be physically active during leisure time [94]. Further, financial instability in Native Americans increases the risk of hypertension by 88% [95]. Therefore, increased financial strain in the Native American communities is exacerbating risk factors for CVD and contributing to the disproportionate burden of CVD.

### 3.3. Healthcare Stressors

Healthcare illiteracy is the lack of fluency with language commonly utilized in healthcare settings and influences the level of comfort patients feel when interacting with physicians and other healthcare professionals. Native American communities have high rates of healthcare illiteracy, which is associated with worse health outcomes [96]. One study reported that 48% of Native American adults have limited healthcare literacy, compared with 36% of all U.S. adults [87]. Despite the lack of research linking healthcare literacy or discrimination in the healthcare setting specifically with CVD, research has linked overall stress and anxiety with an increased risk for CVD [97].

Previous and current discrimination against Native Americans in the healthcare setting also acts as a significant barrier to receiving appropriate and timely healthcare. Much of this discrimination can be attributed to healthcare professionals not exhibiting cultural competency when working with Native American patients [98]. The ability of a healthcare worker to appropriately interact with patients from different backgrounds than their own and to respect the patient’s cultural beliefs greatly impacts the quality of care a patient receives. In a sample of Native American adults with type 2 diabetes mellitus, 36% reported they had experienced microaggressions from their healthcare providers [13]. Microaggressions include negative comments about a person’s background or portray stereotypes that can ultimately minimize a patient’s experience which can have negative impacts on their health [99]. Experiencing microaggressions is positively correlated with a history of heart attacks [99]. Poor experiences in a healthcare setting may deter a patient from receiving care again and could contribute to worse outcomes of CVD.

## 4. Physiological Links Between Chronic Stress and Cardiovascular Health

High levels of chronic stress from historical, financial, and healthcare struggles are apparent in the Native American community and it has been shown that increased stress is correlated with an increased risk for CVD [79,97,100,101]. The INTERHEART study found elevated psychosocial stress over the past year was associated with an over two-fold increased risk for myocardial infarction [102]. As a population endures high levels of chronic stress, it is important to consider and understand the connection between stress and cardiovascular health. The exact mechanisms by which chronic stress affects cardiovascular physiology are not fully understood, but many studies have led to the development of several hypotheses [79]. Addressing these links could provide the ability to improve the physiological response to stress and potentially lower the rate of CVD.

### 4.1. Autonomic Nervous System Dysfunction

The autonomic nervous system (ANS) innervates nearly all the organs of the body and is activated within seconds after exposure to a stressor. The two balancing systems of the ANS are the sympathetic nervous system (SNS) and the parasympathetic nervous system (PNS). The SNS is responsible for the fight-or-flight response by releasing epinephrine from the adrenal medulla and norepinephrine from the sympathetic nerve terminals [103]. The release of these catecholamines promotes optimized blood flow to muscle tissues by increasing heart rate, myocardial contractility, and vascular tone [103]. The PNS functions to inhibit the sympathetic nervous system and predominates during resting conditions.

However, chronic stress results in autonomic dysregulation characterized by sustained sympathetic overactivity and reduced parasympathetic activity. Studies have shown individuals experiencing higher levels of chronic stress have impaired autonomic regulation of cardiovascular functions leading to increased blood pressure, increased variability in heart rate, and reduced ANS sensitivity to acute stressors. Blunted reactivity of the ANS can be analyzed via salivary alpha-amylase, a marker of SNS activation, which is reduced in chronically stressed individuals following an acute mental stressor [104]. An increase in autonomic dysfunction following chronic stress is associated with increased risk for CVDs such as arrhythmias, thrombosis, acute coronary syndromes, and heart failure [105].

Few studies have been conducted assessing the ANS in the Native American population. In Pima Indians, lower SNS activity was associated with a high prevalence of obesity [106]. Further, in Native American adults, a history of alcohol use disorders was associated with low autonomic control and increased hypertension [107]. It is unclear if the autonomic dysregulation is directly caused by chronic stress or if chronic stress may be altering health behaviors such as increased overeating, reduced physical activity, and increased alcohol consumption, resulting in autonomic dysregulation. However, overall, these findings suggest that blunted reactivity of the ANS could contribute to CVD risk in Native Americans.

### 4.2. Hypothalamic–Pituitary–Adrenal Axis Disruption

The hypothalamic–pituitary–adrenal (HPA) axis also acts as the human body’s stress response system and functions to resolve acute stressors [108]. Acute stressors are infrequent and stimulate the hypothalamus to release corticotropin-releasing hormone (CRH) and vasopressin. CRH stimulates the release of adrenocorticotropin hormone (ACTH) from the anterior pituitary which induces the release of glucocorticoids, like cortisol, from the adrenal cortex. One of the functions of cortisol is to maintain control of the HPA axis activation via negative feedback. Cortisol also regulates the production of the glucocorticoid dehydroepiandrosterone (DHEA) which reduces inflammation and protects the brain from damage due to increased cortisol levels. However, chronic stress results in frequent and prolonged activation of the HPA axis marked by hypercortisolism [104]. High levels of cortisol can lead to increased adiposity, insulin resistance, and hypertension, which are known risk factors for CVD [109].

Interestingly, ACTH and cortisol levels are comparable between Pima Indians and whites, suggesting that chronic stress does not lead to hypercortisolism in Native Americans [106,110]. Research indicates that early childhood stress can result in a blunted physiological response to acute stress [111]. This is supported by findings showing that when Pima Indians were administered acute cortisol, their SNS activity decreased—an effect opposite to that observed in whites [110]. Blunted cardiovascular responses to acute physiological stress are associated with adverse health outcomes, including an increased risk of diabetes and higher mortality rates in patients with heart failure. Additionally, it has been suggested that blunted stress reactivity may indirectly contribute to poor cardiovascular outcomes through an increase in adverse health behaviors, such as substance use and overeating, which can lead to addiction and obesity [111]. Further studies are needed to confirm these findings and establish a clearer connection between blunted stress reactivity, adverse health behaviors, and cardiovascular outcomes in the Native American population.

### 4.3. Systemic Low-Level Inflammation

Another key consequence of chronic stress is immune system dysregulation. Cortisol levels following chronic stress are associated with an increase in tumor necrosis factor α (TNF-α) and interleukin 6 (IL-6), markers of low-level inflammation and predictors of cardiovascular health. TNF-α induces the release of IL-6, which is a known factor that links stress and CVDs by promoting vascular damage and the development of atherosclerosis [112].

In addition, studies have shown lower socioeconomic status is inversely correlated with concentrations of other markers of chronic levels of inflammation, such as fibrinogen [113]. Fibrinogen expression can be induced by IL-6 and is an important molecule in both inflammatory and coagulation pathways, leading to an increased risk of thrombosis and acute coronary events [114,115,116]. The production of C-reactive protein (CRP) is also induced by IL-6 and triggers the coagulation pathway by inducing monocytes to express tissue factor [117]. Increased CRP levels can have deleterious effects on the cardiovascular system, demonstrated by the increased risk of recurrent ischemia or myocardial infarction [118,119].

CRP has been established as an independent predictor of cardiovascular events [120]. Additionally, findings from Phase II of the Strong Heart Study demonstrated that elevated levels of CRP and fibrinogen in Native Americans were associated with an increased risk of heart failure [121]. These findings suggest that systemic inflammation may play a significant role in the heightened burden of CVD observed in the Native American population.

### 4.4. Oxidative Stress

In vivo studies have found that chronically stressed rats showed increased levels of oxidative stress [122,123]. Oxidative stress occurs when there is an excess of free radicals, or reactive oxygen species (ROS), that the body cannot neutralize due to a shortage of antioxidants [124]. Chronic inflammatory pathways triggered by prolonged stress elevate TNF-α levels, which promote ROS production [125]. Oxidative stress contributes to platelet hyper-reactivity by exposing a pro-thrombotic environment that triggers platelet activation, which, in turn, generates additional ROS, perpetuating a cycle of oxidative stress [126]. Elevated ROS levels further amplify inflammatory pathways, leading to endothelial dysfunction, and resulting in an increased risk for CVDs such as thrombosis and atherosclerosis [124].

## 5. Discussion

There are a multitude of factors that influence an individual’s risk for CVD. The classical and well-understood modifiable risk factors include diet, exercise, and substance use. We recognize that this review provides only a limited discussion of lipids and atherosclerotic CVD, a gap that reflects the scarcity of research on lipid-related factors in this population and underscores the need for future studies in this area. When considering modifiable factors for CVD, it is crucial to simultaneously evaluate the effect culture may have on them. In Native American communities, there are many social structures that differ from the Western culture and are necessary to consider when trying to understand the increased burden of CVD in Native American populations. These factors are deeply entwined in the culture, so cultural competency is the first step to addressing the cardiovascular disparities in the communities. With a better understanding and appreciation for cultural differences, adequate resources and programs can be created to improve food availability, wellness facilities, education, healthcare access, and programs to reduce substance use.

Other risk factors, such as historical trauma, are more difficult to modify as they are deeply rooted in Native American history and poorly understudied, but may equally contribute to Native Americans’ risk for CVD. Much of this relates to the chronic stress Native Americans may experience due to systemic discrimination and burdens from previous generations. Continued studies of the Native American population are needed to assess the unique mechanisms that have led to increased incidence of CVD compared to the general population, resulting in high levels of morbidity and mortality. The sparse number of published studies has resulted in a limited understanding of the unique pressures, both physiological and environmental, that drive the pathophysiology of the cardiovascular system in the Native American population. Several cellular processes have been identified that are critical for the translation of chronic stress responses to tissue damage resulting in CVDs. Analyzing these pathways and biomarkers in Native American populations could be used to determine if the increased levels of chronic stress in Native Americans contribute to the increased burden of CVD.

Another promising area for future research is platelet reactivity, as thrombosis resulting from platelet hyper-reactivity underlies the pathophysiology of many cardiovascular diseases. Platelet reactivity can be influenced by factors such as diet, cholesterol levels, and smoking [127], and is further exacerbated by conditions like obesity, diabetes, and chronic stress [128,129]. Recent findings have demonstrated, for the first time, that platelets from Native Americans exhibit heightened reactivity compared to those from whites [130]. However, additional studies are needed to explore whether this increased platelet reactivity is associated with specific CVD risk factors within the Native American population.

Due to the historical under-representation of this population in basic and clinical studies, there remains a significant gap in our understanding of Native American health, especially in cardiovascular function. Future studies focused on delineating the underlying pathophysiology of cardiovascular health in Native Americans will aid in the targeting and development of pharmacological, societal, and environmental strategies to limit morbidity due to cardiovascular risk in the Native American population.

## Data Availability

Not applicable.

## Abbreviations

BMI = body mass index, CHD = coronary heart disease, CVD = cardiovascular disease, HDL-C = high-density lipoprotein cholesterol, LDL-C = low-density lipoprotein cholesterol, TG = triglycerides
